# Supplementary material for: Adiponectin, Leptin, and Resistin Are Dysregulated in Patients Infected by SARS-CoV-2
Source: Int J Mol Sci. 2023 Jan 6;24(2):1131. doi: 10.3390/ijms24021131 (PMC9861572; doi:10.3390/ijms24021131)
Supplement: Supplementary file 1 [file ijms-24-01131-s001.zip › ijms-1971237-supplementary.pdf]

Supplementary Table S1

Model Coefficients - ADIPONECTIN (ug/ml)

Predictor	Estimate	SE	t	p
BMI	0.05670	0.1545	0.367	0.714
Glucose	0.00334	0.0106	0.314	0.754
COL. TOT	0.03151	0.0153	2.060	0.042
TRIGLICERIDI	-0.00889	0.0122	-0.728	0.468
COVID	-5.91343	1.6960	-3.487	<.001

[a] Represents reference level

Supplementary Table S2

Model Coefficients - RESISTIN (pg/mL)

Predictor	Estimate	SE	t	p
BMI	-32.89	45.67	-0.720	0.474
Glucose	-6.08	3.20	-1.902	0.061
Total cholesterol	-3.68	5.22	-0.706	0.483
Triglycerides	4.61	4.11	1.123	0.265
COVID	2052.52	575.22	3.568	<.001

[a] Represents reference level

Supplementary Table S3: Comparison of biochemical parameters between discharged (n.47) and death or Intensive outpatient therapy (IOT) (n.15) COVID-19 patients. Data are expressed as median value and interquartile range (IQR).

	DISCHARGED (n.47)			DEATH or IOT (n.15)			TOTAL (n.62)			*p value*
	Median	*25th percentile*	*75th percentile*	*Median*	*25th percentile*	*75th percentile*	*Median*	*25th percentile*	*75th percentile*	
WBC	8.79	6.86	11.7	11.3	7.49	13.3	9.01	6.85	12.2	0.241
Neutrophils	7.75	5.26	10.6	10.4	6.1	11.6	7.98	5.61	10.7	0.276
Lymphocytes	0.805	0.57	1.13	0.63	0.43	1.1	0.73	0.57	1.14	0.37
NLR	10.5	5.45	14.6	10.9	8.58	15	10.8	5.82	14.8	0.473
RBC	4.72	4.55	5.25	4.5	4.24	5.07	4.7	4.5	5.15	0.096
HGB	13.7	12.5	14.3	12.7	11.8	13.7	13.4	12.3	14.3	0.125
HCT	39.5	37.3	41.2	37.3	34.6	40.4	39.2	36.4	41	0.241
PLT	232	188	280	230	171	299	232	183	295	0.575
INR	1.13	1.07	1.2	1.1	1.06	1.25	1.13	1.07	1.21	0.621
PT	82.5	75	90.5	86	72	92	83	75	91	0.615
aPTT	28	25.1	31.5	27.5	25.9	33.5	27.9	25.2	32	0.749
D-dimer	281	177	519	569	425	1723	402	208	651	**0.007**
Fibrinogen	641	585	707	571	536	711	641	563	708	0.589
Azotaemia	52	43	67	61	53.5	86	55	44	68	0.062
Creatinine	0.7	0.6	0.9	0.8	0.6	1	0.75	0.6	0.9	0.678
Na+	138	135	140	140	136	142	138	135	140	0.244
K+	4.1	3.77	4.63	4.1	3.65	4.5	4.1	3.7	4.6	0.875
AST	35	25.5	52	35	27	52.5	35	26	52.5	0.773
ALT	32	22.5	70.5	32	23	66.5	32	21.8	70	0.844
LDH	324	273	421	509	341	635	341	289	494	**0.003**
PCR	8.2	4.8	12.1	7	2.75	12.1	7.8	3.85	12.3	0.628
Triglycerides	157	126	212	147	122	153	150	123	203	0.814
GGT	41	30	89	71.5	36	86.8	49	30	88	0.711
Alkaline phosp	53	50	76	62	43	76	55.5	47.8	76	0.814
Amylasis	84	62	106	67	57	68	67.5	58.3	105	0.724
Lipasis	44	31	55	53	38	57	44.5	31.5	56.5	0.906
Total Bilirubin	0.64	0.49	0.905	0.755	0.578	0.915	0.68	0.51	0.91	0.233
Albumin	4.2	3.73	4.3	3.6	3.23	4.13	4.15	3.38	4.3	0.136
IL 6	1022	720	1420	1061	921	1808	40.2	17.5	72.1	**0.048**
IL2R	28.4	16.2	55.5	53.8	42.1	137	1061	794	1464	0.281
KL6	513	436	786	1810	800	2464	552	467	1053	**<.001**
Adiponectin (µg/ml)	14.7	11.9	16.3	15.1	13.9	18	14.7	13.1	16.4	0.393
Leptin (ng/ml)	17.4	7.09	25.5	15.8	8.87	20.8	16.6	7.79	25.1	0.536

Adiponectin/Leptin	0.762	0.599	2.18	0.944	0.706	1.79	0.823	0.618	1.99	0.274
Resistin (pg/mL)	5613	4341	6935	5798	4657	7403	5645	4395	7177	0.606

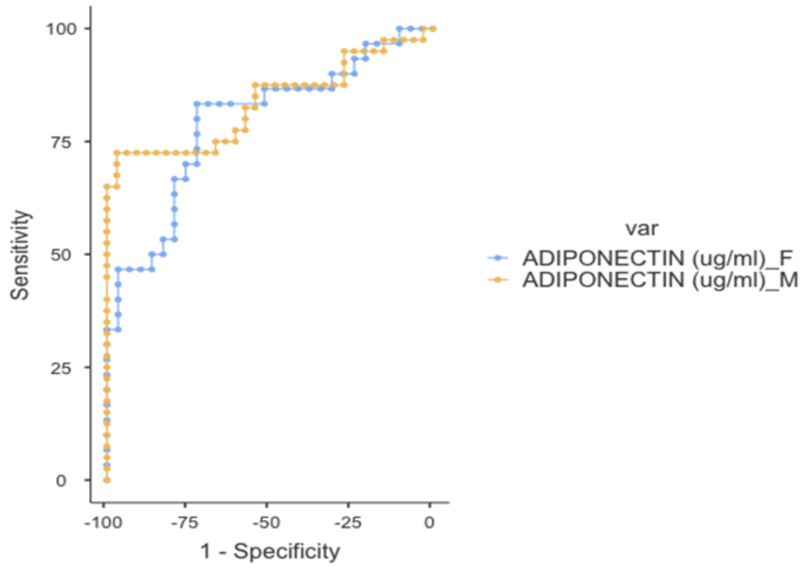

Scale: ADIPONECTIN (ug/ml)_Male

Cutpoint	Sensitivity (%)	Specificity (%)	PPV (%)	NPV (%)	Youden's index	AUC	Metric Score
18.023 05476	70.27%	96.97%	96.3%	74.42%	0.672	0.822	1.67

Scale: ADIPONECTIN (ug/ml)_Female

Cutpoint	Sensitivity (%)	Specificity (%)	PPV (%)	NPV (%)	Youden's index	AUC	Metric Score
15.788 96698	80%	72.41%	71.43%	80.77%	0.524	0.750	1.52

Figure S1

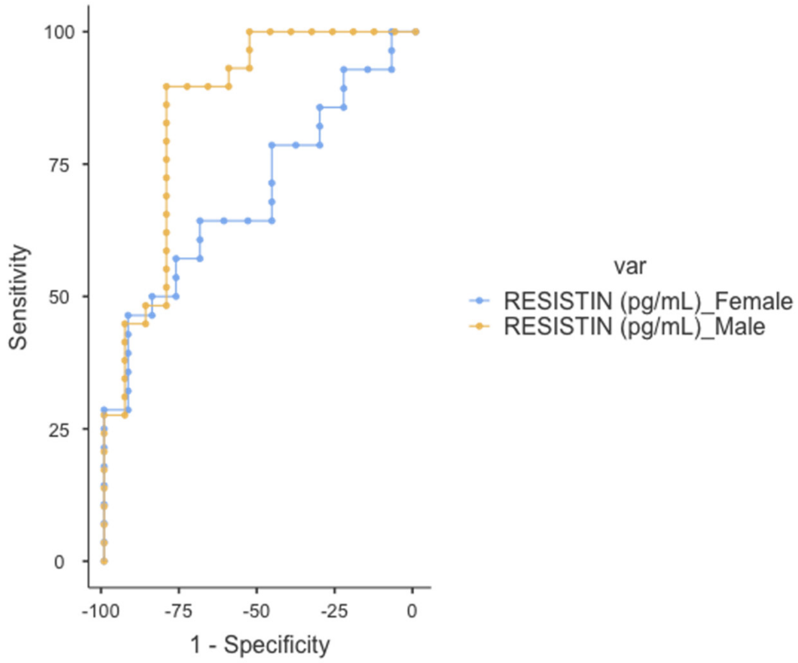

Scale: RESISTIN (pg/mL)_Male

Cutpoint	Sensitivity (%)	Specificity (%)	PPV (%)	NPV (%)	Youden's index	AUC	Metric Score
4322.6	89.66%	80%	89.66%	80%	0.697	0.855	1.70

Scale: RESISTIN (pg/mL)_Female

Cutpoint	Sensitivity (%)	Specificity (%)	PPV (%)	NPV (%)	Youden's index	AUC	Metric Score
4838.7	57.14%	76.92%	84.21%	45.45%	0.341	0.695	1.34

Figure S2
